# SUMOs Mediate the Nuclear Transfer of p38 and p-p38 during *Helicobacter Pylori* Infection

**DOI:** 10.3390/ijms19092482

**Published:** 2018-08-22

**Authors:** Pin Yao Wang, Ping I. Hsu, Deng Chyang Wu, Te Chung Chen, Andrew Paul Jarman, Lynn Marie Powell, Angela Chen

**Affiliations:** 1 Institute of Biomedical Sciences, National Sun Yat-Sen University, Kaohsiung 80424, Taiwan; M922050002@gmail.com (P.Y.W.); dino6361@gmail.com (T.C.C.); achen@mail.nsysu.edu.tw (A.C.); 2 Division of Gastroenterology, Department of Internal Medicine, Kaohsiung Veterans General Hospital, Kaohsiung 81362, Taiwan; pihsu@vghks.gov.tw; 3 Division of Gastroenterology, Department of Internal Medicine, Kaohsiung Municipal Ta-Tung Hospital, Kaohsiung Medical University Hospital, Kaohsiung Medical University, Kaohsiung 80708, Taiwan; gi@kmu.edu.tw; 4 Centre for Discovery Brain Sciences, School of Biomedical Sciences, University of Edinburgh, George Square, Edinburgh EH8 9XD, UK; andrew.jarman@ed.ac.uk; 5 Institute of Cell Biology, School of Biological Sciences, University of Edinburgh, King’s Buildings, Roger Land Building, Alexander Crum Brown Road, Edinburgh EH9 3FF, UK

**Keywords:** *Helicobacter pylori*, nuclear transfer, p38, signal pathway, SIM, SUMO-2

## Abstract

The p38 mitogen activated protein kinase (MAPK) signaling pathway has been suggested to play a significant role in the gastric mucosal inflammatory response to chronic *Helicobacter pylori* (*H. pylori*) infection. Nuclear translocation is thought to be important for p38 function, but no nuclear translocation signals have been found in the protein and no nuclear carrier proteins have been identified for p38. We have investigated the role of small ubiquitin-related modifier (SUMO) in the nuclear transfer of p38 in response to *H. pylori* infection. Exposure of human AGS cells to *H. pylori* induced the activation of p38 and the expression of SUMOs, especially SUMO-2. SUMO knockdown counteracted the effect of *H. pylori* infection by decreasing the resulting p38 mediated cellular apoptosis through a reduction in the nuclear fraction of phosphorylated p38. We identified a non-covalent interaction between SUMOs and p38 via SUMO interaction motifs (SIMs), and showed that SUMO-dependent nuclear transfer of p38 was decreased upon mutation of its SIMs. This study has identified a new pathway of p38 nuclear translocation, in response to *H. pylori* infection. We conclude that in the presence of *H. pylori* SUMO-2 has a major role in regulating nuclear levels of p38, through non-covalent SUMO-p38 interactions, independent of the p38 phosphorylation state.

## 1. Introduction

Environmental insults such as oxidative stress and hypoxia can induce the activation of the p38 mitogen activated protein kinase (MAPK) signaling pathway, causing a variety of cellular responses such as apoptosis [1,2]. p38 MAPK is activated following phosphorylation at Thr180/Tyr182 within the activation loop (p-p38), primarily by upstream MKK3 and MKK6 [3]. p38 MAPK has been shown to be distributed throughout the cytosol and nucleus [4]. Phosphorylation-dependent nuclear translocation of p38 has been reported to be a common phenomenon when cells are stimulated by various stresses [5]. However, no nuclear translocation signals (NTSs) have been found in p38 and no nuclear carrier proteins have been identified for it. It is therefore unclear how nuclear translocation of p38 is achieved [6,7,8,9].

*Helicobacter pylori* (*H. pylori* or *Hp*) is thought to induce gastric epithelial inflammation and apoptosis, and can interfere with the ulcer healing process within the stomach. Oxidative stress and cellular apoptosis have been observed in *H. pylori* infected gastric tissue, which may be due to inflammation caused by overproduction of cytokines stimulated by the infection [10,11]. The p38 MAPK signaling pathway has been suggested to play a significant role in the gastric mucosal inflammatory response to chronic *H. pylori* infection via prostaglandin E_2_ [12]. MAPK activation, particularly via JNK and p38, is more potently induced by Cag^+^ compared with Cag^−^ strains of clinical *H. pylori* [13]. The toxin Vac-A of Vac^+^ *H. pylori* strains may induce apoptosis through differential regulation of ERK1/2 and p38 MAPK [14].

The small ubiquitin-related modifier (SUMO), an important post-translational modifier, has been implicated in a wide range of cellular processes including intracellular targeting, response to extracellular stimuli, transcriptional regulation, differentiation, cytoplasmic to nuclear translocation, and apoptosis [15,16,17,18,19]. SUMO-1 has a major role in the formation of promyelocytic leukemia nuclear bodies (PML-NBs), which appear in response to viral infections [20] and environmental stresses, including oxidative stress [21]. When cells were subjected to protein-damaging stimuli via heat shock and ethanol addition, resulting in oxidative stress, large quantities of free, non-conjugated SUMO-2 were produced and high levels of SUMO-2 conjugates were detected. Under such stresses SUMO-2 was found to be more abundant than SUMO-1 [16]. SUMO has previously been shown to be important for nuclear transport of certain proteins not only by covalent modification but also by non-covalent interaction. For example, the SAE2 subunit of human SUMO activation enzyme has been shown to be dependent on SUMOylation at its C terminus for nuclear localization [22]. In contrast non-covalent association of parkin with SUMO-1 results in an increase in the nuclear transport of parkin [15]. In addition, our previous study showed that although Daxx protein usually depends on a nuclear localization signal (NLS) for transport from the cytoplasm to the nucleus, NLS mutated Daxx can be transferred from the cytoplasm to the nucleus by utilizing SUMOs as carrier proteins in co-expressing cells [18]. It has previously been shown that SUMOs may have differing binding affinities for various substrates; e.g., TNF receptor-associated protein (TRAF) preferentially binds to SUMO-2 whilst Ran binding-protein 2 (RanBP2) preferentially binds to SUMO-1 [23], and Bloom syndrome protein binds SUMO-2 in preference to SUMO-1 [24]. GST-Daxx has previously been observed to be strongly modified by SUMO-1 and weakly modified by SUMO-2 [25]. In this study we have found that SUMOs (in particular SUMO-2) were upregulated in AGS cells in response to *H. pylori* infection, in parallel with p38 activation. Therefore, SUMO-1 and SUMO-2 were examined for their roles in nuclear translocation of p38. Here we show that SUMO-2 mediates *H. pylori* induced p38-dependent apoptosis via the translocation of p38 to the nucleus in response to *H. pylori* infection.

## 2. Results

### 2.1. The Association between Up-Regulation of SUMOs and Activation of the p38 Pathway, in Response to H. pylori Infection

**Figure 1:**
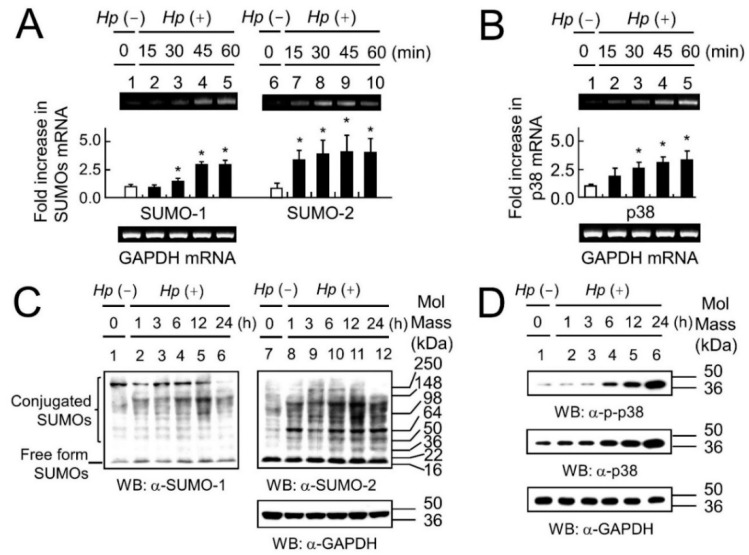
*H. pylori* infection induces the expression of small ubiquitin-related modifier (SUMO)-1, SUMO-2, p38 and p-p38 in AGS cells. Total RNA was isolated from AGS cells infected with *H. pylori* for 15, 30, 45 and 60 min (black bars). The mRNA expression levels of SUMO-1, SUMO-2, p38 and GAPDH were obtained by reverse transcription polymerase chain reaction (RT-PCR). The mRNA expression levels of (**A**) SUMO-1 and SUMO-2 and (**B**) p38, were up-regulated with increasing periods of time during *H. pylori* infection, as indicated, normalized to the mRNA levels observed in uninfected cells (white bar). Each experiment in (**A**,**B**) was repeated at least three times and all data are represented in the plots as the mean ± the standard deviation. Differences between each group and the *Hp* (−) condition were evaluated by an independent Student’s *t*-test using SPSS version 20.0 (SPSS Inc., Chicago, IL, USA). *p* < 0.05 (*) was considered significant. Representative gels are shown in each case. Western blot analysis showed that the protein expression levels of SUMO-1 and SUMO-2 (**C**; lanes 2–6 and lanes 8–12), and p-p38 and p38 (**D**; lanes 2–6), were up-regulated with increasing periods of time following *H. pylori* infection for 1, 3, 6, 12 and 24 h compared with those in the absence of *H. pylori* infection in Lanes 1 and 7. In a separate experiment (Figure S1B) early upregulation of p-p38 was seen after only 45 min of *H. pylori* infection. The early upregulation is not apparent here (**D**; 1 h) as the exposure time was limited to avoid saturation at later time points. In the case of SUMOs the increase is apparent in the conjugated forms of the proteins, and SUMO-2 up-regulation was greater than that of SUMO-1 in response to *H. pylori* infection (**C**). The experiments in (**C**,**D**) were repeated three times and representative images are shown.

Previous studies have shown that SUMOs are increased in response to various stresses [16,26,27] and that p38 mRNA and protein are increased in response to *H. pylori* infection or in response to the *H. pylori* cytotoxins VacA and CagA [13,14,28], hence our first steps were to measure SUMOs and p38 mRNAs and proteins in response to *H. pylori* infection. We chose the strongly virulent *H. pylori* strain ATCC 43504 (*CagA*^+^, *VacA*^+^) for our studies, as less virulent strains (e.g., CagA negative strains) would be expected to produce a weaker p38 response. The mRNA levels of SUMO-1 and SUMO-2 (Figure 1A), and p38 (Figure 1B) were up-regulated at increasing periods of time during *H. pylori* infection. Similarly, increased protein expression levels for SUMO-1 and SUMO-2 (Figure 1C), as well as p38 and p-p38 (Figure 1D) were seen in response to chronic *H. pylori* infection over a period of 24 h. A significant increase in the activated form of p38 (p-p38) was also seen after shorter periods of *H. pylori* infection (Figure S1B and Table S1B) although total p38 did not increase under these conditions. Similar early induction of p-p38 one hour after *H. pylori* infection has been shown previously [13]. It has also previously been shown that a large pool of free, non-conjugated SUMO-2 was induced in response to various stresses and that SUMO-2 was more abundant than SUMO-1. In this study, induction of high levels of SUMOs under the infective stress of *H. pylori* infections was reflected in the increase in the total conjugated forms of SUMOs, and in agreement with previous studies SUMO-2 was more abundant than SUMO-1 (Figure 1C).

Hence, our results and those documented previously showed that p38-MAPK signaling increased in the presence of *H. pylori* infection. Previous papers showed that SUMOs increased in response to various stressors, and we have demonstrated that SUMOs, and in particular SUMO-2 increased in response to *H. pylori* infection (Figure 1C). p-p38 must be transported to the nucleus to exert its effects, and a well-known role of SUMOs is to alter sub-cellular localization. Therefore, we decided to see whether there was a link between SUMOs and p38 activity (namely p38-mediated apoptosis), and also between SUMOs and p38 localization within the cell.

**Figure 2:**
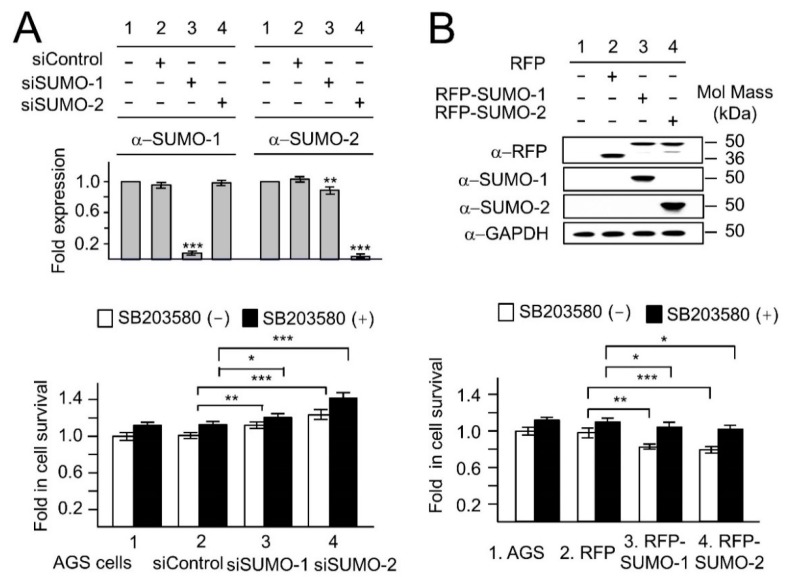
SUMOs-dependent reductions in cell survival during *H. pylori* infection arise due to p38-mediated apoptosis. The levels of *H. pylori* induced apoptosis for various transfectants (**A**) siSUMO-1, siSUMO-2, siControl, and (**B**) RFP-vector, RFP-SUMO-1 and RFP-SUMO-2 were examined in AGS cells by MTT assays, in which SB203580 (p38 MAPK inhibitor) was used to assess the role of the p38-mediated apoptotic pathway. (**A**) Upper panel: The knockdown of SUMO-1 and SUMO-2 by siSUMO-1 and siSUMO-2 respectively was confirmed by quantification of Western blot data. We also noticed a small but significant non-specific reduction of SUMO-2 by si-SUMO-1. Lower panel: The increased cell survival in each group of SB 203580 pretreated cells (black bars vs. white bars; see Table S2 for summary of statistical analyses) during *H. pylori* infection showed that p38 was involved in *H. pylori* induced apoptosis. Cell survival was significantly increased in siSUMO-1 and siSUMO-2 transfectants (siSUMO-1 or siSUMO-2 vs. siControl) with or without SB203580. (**B**) Upper panel: The expression of RFP, RFP-SUMO-1 and RFP-SUMO-2 respectively was confirmed by Western blotting. Lower panel: In contrast to the si-SUMOs data, cell survival decreased in RFP-SUMO-1 and RFP-SUMO-2 expressing cells (RFP-SUMO-1 or RFP-SUMO-2 vs. RFP). This effect was reversed by the use of the p38 MAPK inhibitor SB203580 (compare black bars to white bars in groups 3 and 4), showing that the effect of SUMOs depends on activated p38. This is consistent with SUMOs reducing cell viability via the p38-mediated apoptotic pathway. * *p* < 0.05; ** *p* < 0.01; *** *p* < 0.001. The Western blots shown in the upper panels were repeated three times and the results shown in (**A**) are the mean ± standard deviation of these three repeats. In (**B**) a representative image of the three repeats is shown. The MTT assays in the lower panel were repeated 4 times for each condition and the results shown are the mean ± standard deviation. All data were evaluated by independent Student’s *t*-test using SPSS version 20.0 (SPSS Inc., Chicago, IL, USA). *p* < 0.05 was considered significant. The *p* values obtained for the data shown in (**A**,**B**) have been summarized in Table S2.

In order to examine the influence of SUMO on the p38-mediated apoptotic pathway in response to *H. pylori* infection, the MTT assay for cell survival was performed in siSUMO and RFP-SUMO transfectants with or without p38-MAPK inhibitor SB203580 (Figure 2). Cell survival levels were significantly higher upon inhibition of the p38-MAPK pathway in SB203580 pretreated cells in each case (Figure 2, black bar vs. white bar; see also the summary of statistical analyses in Table S2).

Quantification of Western blot data showed the decrease in the levels of endogenous SUMO-1 and SUMO-2 in siSUMO-1 and siSUMO-2 transfectants, respectively (Figure 2A, top panel), while RFP-SUMO-1 and RFP-SUMO-2 fusion proteins were used to increase levels of SUMO-1 and SUMO-2 (Western blot in Figure 2B, top panel). We also observed that siSUMO-1 produced a small but significant decrease in SUMO-2 levels. This is consistent with a non-specific effect of siSUMO-1 on SUMO-2. The knockdown of SUMO-1 and SUMO-2 increased cell survival in *H. pylori* infected AGS cells (Figure S1A, groups 3 and 4 vs. group 2; Table S1; and Figure S2A, Lower panel, groups 3 and 4 vs. group 2). In contrast comparisons between RFP-SUMO-1 or RFP-SUMO-2 transfectants with the RFP control transfectants (Figure 2B, lane 3 and lane 4 vs. lane 2 respectively) showed that overexpression of SUMOs lead to a significant decrease in cell survival in RFP-SUMOs expressing cells. This effect was partially rescued when SUMOs overexpression was accompanied by treatment with the p38 pathway inhibitor SB203580, consistent with at least part of the SUMOs effect being via p38-mediated apoptosis. This incomplete rescue can be explained firstly, because p38-mediated apoptosis is not the only apoptotic pathway to be induced during *H. pylori* infection [13] we cannot rule out an effect of SUMOs on such alternative pathways too. Secondly, although SB203580 is the most effective inhibitor for the p38 pathway it would not be expected to completely block it. We note that in addition to the large knockdown of SUMO-1 by si-SUMO-1, the small but significant knockdown of SUMO-2 by si-SUMO-1 may contribute to the increased cell survival in this case. However, the misexpression of SUMO-1 and SUMO-2 both resulted in decreased cell survival confirming that SUMO-1, like SUMO-2, is capable of affecting cell survival via p38-mediated apoptosis.

With respect to the p38 pathway we found that overexpression of SUMOs enhanced endogenous p38 phosphorylation as shown in Figure S1B (see also Table S1 for statistical analyses), and SUMOs were found to colocalize with p-p38 in nuclear dots in cells overexpressing SUMOs as shown in Figure S2. Taken together these results demonstrate that the levels of SUMOs in cells have an impact on p38 mediated apoptosis possibly via direct interaction between SUMOs and p38.

### 2.2. The Nuclear Localization of p38 and p-p38 Is Dependent on the Levels of SUMOs

**Figure 3:**
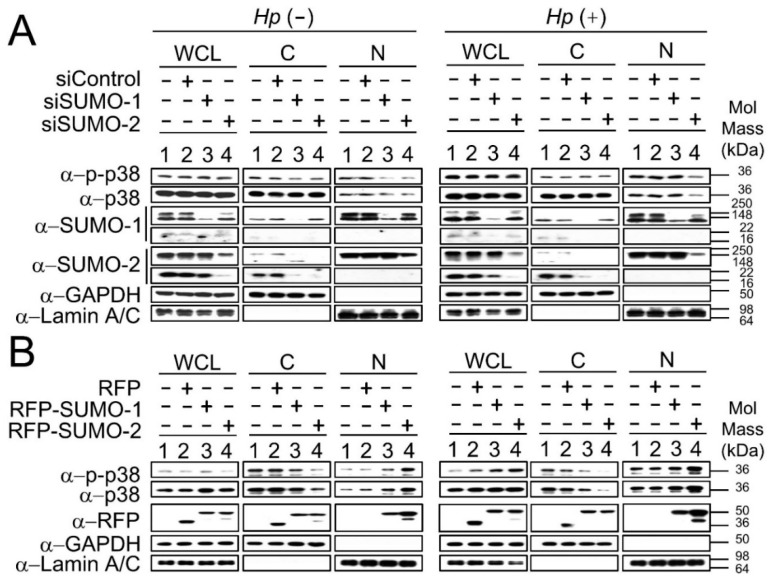
SUMO-2 is more efficient than SUMO-1 in regulating nuclear p38 and p-p38 during *H. pylori* infection. The cytoplasmic and nuclear fractions of p38 and p-p38 were analyzed from siSUMO transfectants (**A**) and RFP-SUMO transfectants (**B**). p38 and p-p38 were blotted with anti-p38 and anti-p-p38 antibodies. SUMO-1 and SUMO-2 were detected using anti-SUMO-1 and anti-SUMO-2 antibodies. RFP alone or fusion proteins of RFP-SUMO-1 or RFP-SUMO-2, were detected using anti-RFP. GAPDH and Lamin A/C were used as cytosolic and nuclear markers respectively. For α-SUMO-1 and α-SUMO-2 the upper panels show conjugated SUMOs while the lower panels show free-form SUMOs. (**A**) Western Blots showed that the endogenous SUMO-1 and SUMO-2 were down-regulated after transfectional incubation of siSUMO-1 and siSUMO-2. p38 and p-p38 in the N-fraction were decreased in siSUMO-1 and siSUMO-2 transfectants without *H. pylori* infection; however, their nuclear levels decreased only for siSUMO-2 and not for siSUMO-1 transfectants during *H. pylori* infection; and (**B**) Western Blots showed that RFP-SUMO-1 and RFP-SUMO-2 fusion proteins were up-regulated after transfectional incubation. p38 and p-p38 levels were up-regulated in the N-fraction and clearly down-regulated in the C-fraction in RFP-SUMO-2 transfectants during *H. pylori* infection. Quantification of the blots shown in (**A**,**B**) is summarized in Table S3 below. All experiments were repeated three times and representative images are shown.

To further investigate the function of SUMOs in relation to p38 proteins during *H. pylori* infection, p38 and p-p38 in nuclear or cytoplasmic subcellular fractions of siSUMO (Figure 3A) and RFP-SUMO (Figure 3B) transfectants were analyzed with or without *H. pylori* infection. In whole cell lysate (WCL) endogenous p-p38 was up-regulated (Figure 3A, right panel, groups 1–4 compared with left panel, groups 1–4) in response to *Hp* infection. Endogenous SUMO-1 (Figure 3A, group 3) and SUMO-2 (Figure 3A, group 4) were down-regulated after transfection of siSUMO-1 and siSUMO-2 with or without *Hp* infection. Without *Hp* infection p38 and p-p38 in the nuclear fraction (N-fraction) were decreased in siSUMO-1 (Figure 3A, left panel, group 3) and siSUMO-2 (Figure 3A, left panel, lane 4) transfectants. This suggests that SUMO-1 and SUMO-2 share the same role in regulation of nuclear levels of p38 and p-p38 when cells are in their resting state (without *H. pylori* infection). However, during *Hp* infection a decrease in levels of p38 and p-p38 in the N-fraction occurred only in siSUMO-2 transfectants (Figure 3A, right panel, group 4), but not in siSUMO-1 transfectants (Figure 3A, right panel, group 3). This result suggests that there may be different mechanisms by which SUMOs regulate nuclear levels of p38 and p-p38 with or without stimulation i.e., both SUMOs have a role in the absence of stimuli, whereas after stimulation it is SUMO-2 that has the major role in transporting p38 to the nucleus. The same nuclear fractionation assay was performed in RFP-SUMO expressing cells (Figure 3B). RFP-vector (RFP) was used as a control for the RFP-fusion fragment without SUMO. In response to *Hp* infection, both p38 and p-p38 proteins were significantly up-regulated in the N-fraction but decreased in the cytoplasmic fraction (C-fraction) in the RFP-SUMO-2 expressing cells (Figure 3B, right panel, group 4) compared to RFP-vector (Figure 3B, right panel, group 2) and RFP-SUMO-1 (Figure 3B, right panel, group 3) transfectants. These results suggest that SUMO-2 has a major role in regulating the nuclear levels of p38 and p-p38 in response to *H. pylori* infection.

### 2.3. SUMO Mediated Nuclear Transfer of p38 and p-p38 Is SUMO Interacting Motif (SIM) Dependent

Our results (Figure 3) showed that SUMO-2 has a major role in regulating the nuclear levels of p38 and p-p38 in response to *H. pylori* infection. In order to explain the association between levels of SUMOs and p38, and the connections between SUMOs and p38-mediated apoptosis in response to *H. pylori* infection, we hypothesized that SUMO may be involved in the nuclear transfer of p38. To investigate this hypothesis, we tested the possibility that a SIM on p38 could be the interaction site. SIMs have been identified in many SUMOylation substrates, such as RanBP2/Nup358 and SUMO ligases PIASX [23,29]. SIMs are characterized by a loose consensus sequence and variants are plentiful. It has become evident that hydrophobic residues are quintessential components of SIMs [30]; hence we mutated hydrophobic residues in three predicted SIMs in p38 and tested the effect on binding to SUMOs.

**Figure 4:**
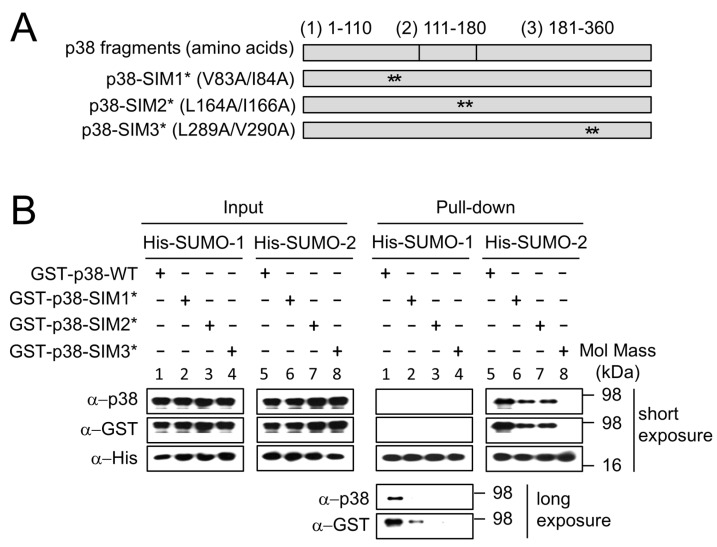
The SUMO binding abilities of p38 were decreased in SUMO interacting motif (SIM) mutants. (**A**) Predicted SIMs from three SUMO-p38 interacting fragments (see Materials and Methods) were individually mutated in the context of full-length p38 to give three full length p38-SIM mutants with two amino acid residues mutated in each; and (**B**) GST-p38-WT and GST-p38-SIM1*, GST-p38-SIM2* and GST-p38-SIM3* expression constructs were used for in vitro SUMOs pull-down assays. Purified His-SUMOs and Ni-NTA-beads were mixed before addition of the target GST-p38-WT and GST-p38-SIM1*, GST-p38-SIM2* and GST-p38-SIM3* fusion proteins followed by incubation to form complexes of bead-conjugated-His-SUMOs and GST-p38. SUMO to p38 ratios of 1:1 were used. The binding affinities of SUMO-2 for all three p38-SIM mutants were decreased compared to wild type, especially for p38-SIM3*. Binding of p38 by SUMO-1 is much weaker than by SUMO-2, and could only be seen for p38-WT after long exposure of the Western blot. All experiments were repeated three times and representative images are shown.

The GST fusion proteins of p38-WT (p38-WT, a full length p38 containing 360 amino acid residues) and the SIM mutants p38-SIM1*, p38-SIM2* and p38-SIM3* (full length p38 with mutations in SIMs1, 2 and 3 respectively) were constructed as shown in Figure 4A and the binding ability to His-SUMO-1 and His-SUMO-2 were analyzed using a pull-down assay. The binding of SUMO-2 to p38-SIM3* was clearly abolished (Figure 4B group 8; Pull-down) and that to GST-p38-SIM1* and GST-p38-SIM2* were significantly decreased (Figure 4B, groups 6-7; Pull-down) compared to that of wild type p38, GST-p38-WT (Figure 4B, group 5; Pull-down). The binding of SUMO-1 to p38 was too weak to detect, unless a longer exposure time was used (Figure 4B groups 1–4; Pull-down; compare the long and short exposures).

**Figure 5:**
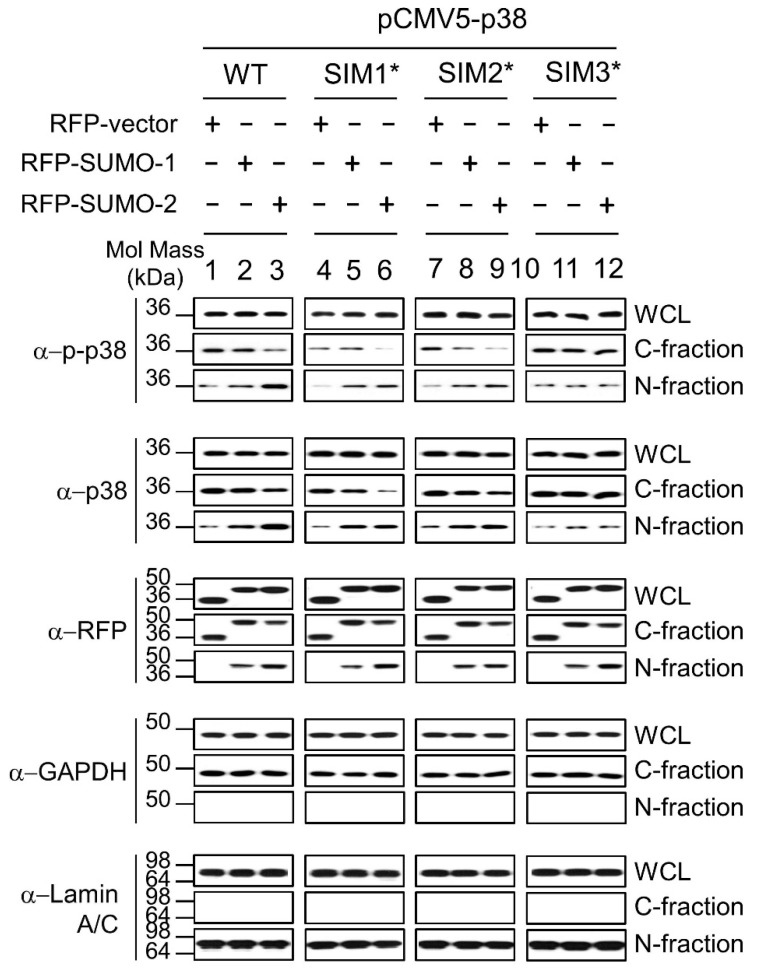
The nuclear transfer of p-p-38 and p38 by SUMOs was decreased for p38 SIM mutants. Cellular fractionation assays during *H. pylori* infection were used to evaluate the nuclear transfer abilities of SUMOs for p38-SIM mutants in AGS cells cotransfected with constructs expressing p38 variants as indicated together with expression constructs for RFP, RFP-SUMO-1 or RFP-SUMO-2. A significant decrease in the nuclear levels of p38-SIM3* and a mild decrease in the nuclear levels of p38-SIM1* and p38-SIM2* were observed compared with p38-WT, in particular in the presence of RFP-SUMO-2, for both p38 and p-p38. All experiments were repeated three times and representative images are shown.

Next, we analyzed these interactions in AGS cells (Figure 5). The cytosolic-fractions (C-fractions) and the nuclear-fractions (N-fractions) of the RFP-vector (negative control for SUMO proteins), RFP-SUMO-1, RFP-SUMO-2, and pCMV5-p38-WT and mutants (pCMV5-p38-SIM1*, pCMV5-p38-SIM2*, pCMV5-p38-SIM3*) co-expressing cells in response to *H. pylori* infection were analyzed as shown in Figure 5. The results demonstrate that, in comparison to p38-WT, increased nuclear transfer of p38 and p-p38 were slightly decreased in RFP-SUMO-2/p38-SIM1* or RFP-SUMO-2/p38-SIM2* co-expression cells (Figure 5: Lane 6 or 9 cf lane 3 in N-fraction panel) and abolished in the RFP-SUMO-2/p38-SIM3* co-expressing cells (Figure 5: Lane 12 vs. 3 in N-fraction panel). Similar effects were observed in RFP-SUMO-1/p38-SIM mutants co-expressing cells, but not as clearly as for SUMO-2. We therefore concluded that SUMOs are involved in mediating the nuclear transfer of both p38 and p-p38 through interactions between SUMOs and the p38 SIMs, and that p38-SIM3 at amino acid residues 289-292 (LVLD) is the major interacting site.

### 2.4. SUMO Interacts with p38 Non-Covalently

**Figure 6:**
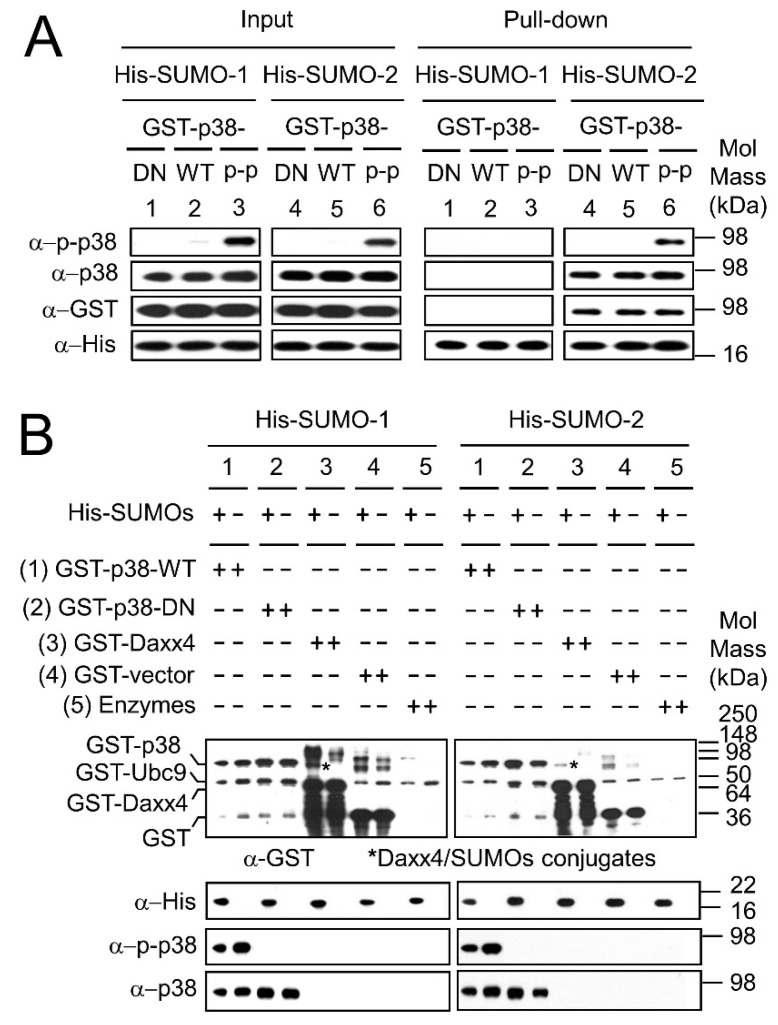
SUMO-2 binds to p38-DN, p38-WT and p-p38 non-covalently. GST-p38-WT and GST-p38-DN expression constructs were used for in vitro SUMOs pull-down assay and SUMOylation assay. GST-p-p38, for the in vitro SUMOs pull-down assay, was obtained by incubating GST-p38-WT with an ATP regeneration system. Purified His-SUMOs and Ni-NTA-beads were mixed before the target GST-p38-WT, GST-p38-DN and GST-p-p38 fusion proteins were added and incubated in order to form complexes of bead-conjugated-His-SUMOs and GST-p38. A SUMO to p38 ratio of 1:1 was used. (**A**) The blots for input proteins and the pull down proteins were probed with antibodies. SUMO-2 can pull-down GST-p38-DN, GST-p38-WT and GST-p-p38, but SUMO-1 is unable to do so at the SUMO to p38 ratio of 1:1; and (**B**) In vitro SUMOylation assays were done as previously described [24] and SUMOylation products were discerned by immunoblot analysis. No newly formed SUMOylation products were observed in the reactions for GST-p38-WT or GST-p38-DN, although SUMOylation products (marked with asterisks) were observed for the positive control GST-Daxx4 (a GST fusion of a tail fragment of Daxx, 607–740 aa, with a total molecular mass of 50 kDa). All experiments were repeated three times and representative images are shown.

Our results (Figure 5) showed that SUMO-2 has a major role in regulating the nuclear levels of both p38 and p-p38 through their SIMs in response to *H. pylori* infection. It has previously been shown that SUMOs may have differing binding affinities for various substrates, and our pull-down results in Figure 4 suggested that the SUMO-2-p38 interaction was stronger than the SUMO-1-p38 interaction, therefore, we next sought to investigate the effect of the p38 phosphorylation state on the binding affinities of SUMO-1 and SUMO-2 to p38 and p-p38. In order to investigate a non-phosphorylatable form of p38 the GST-p38-DN protein expression construct was made, containing full length p38 with the two phosphorylation sites at amino acid residues 180 and 182 mutated. The resulting protein p38-DN functions as a dominant negative protein. Phosphorylated GST-p38-WT (GST-p-p38-WT) was produced by using the ATP regeneration system in the in vitro SUMOylation reactions. His-fused SUMO-1 and SUMO-2 proteins were used to pull-down GST-p38-DN, GST-p38-WT and GST-p-p38-WT proteins to compare the binding affinity to p38 for SUMO-1 versus SUMO-2 (SUMOs to p38 ratio of 1:1) (Figure 6A). Figure 6A shows the input for reactions blotted with anti-p-p38 antibody, anti-p38 antibody, anti-GST antibody and anti-His antibody in the left panel, and the corresponding pull-downs in the right panel. The pull-down results (Figure 6A, right panel, groups 4–6) demonstrate that SUMO-2 pull-down products for GST-p38-DN, GST-p38-WT and GST-p-p38-WT were clearly observed; however, no SUMO-1 pull-down products were observed (Figure 6A, right panel, groups 1–3).

These results confirm that SUMO-2 has a better binding affinity for p38 (regardless of phosphorylation state) compared to SUMO-1. However, when the levels of SUMO to p38 were increased to a ratio of 5:1, both SUMO-2 and SUMO-1 clearly interact with p-p38-WT and with p38-DN (Figure S3). This confirms that in vitro SUMOs interact with both non-phosphorylated and phosphorylated p38 (p38 and p-p38 respectively).

Since interactions between SUMOs and p38 were observed in direct pull-down of p38 and p-p38 by SUMOs, in vitro SUMO modification assays (Figure 6B) were performed to determine whether p38 is a substrate for SUMOylation. GST-p38-WT (group 1) and GST-p38-DN (group 2) were used as SUMOylation substrates, GST-Daxx4 (a Daxx tail fragment fused to GST) functioned as a SUMOylation positive control (group 3); GST only protein (group 4) and SUMOylation enzymes (GST-SAE1/SAE2 and GST-Ubc9) (group 5) were two negative controls as GST-Ubc9 (group 5), although itself a substrate for SUMOylation was at too low a level for us to detect SUMOylation under these assay conditions. Reactions were either in the presence or absence of SUMOs as indicated in Figure 6B. Anti-GST antibody was used to identify the following proteins: GST-p38-WT, GST-p38-DN, GST-Daxx4, GST, GST-Ubc9 and newly formed SUMOylation GST-fusion protein conjugates. p38 variants were detected using anti-p38 and anti-p-p38. Figure 6B shows that except for the positive control GST-Daxx4 (Figure 6B, group 3), no newly formed SUMOylation products were observed in the reactions (comparing His-SUMOs (+) with His-SUMOs (−) lanes). The assay was repeated several times and the blots overexposed [31] in order to check for any indication of SUMOylated p38 species, but none were found. These results suggest that GST-p38-WT and GST-p38-DN are not SUMOylation substrates for SUMO-1 or SUMO-2, consistent with interactions between SUMOs and p38 being predominantly non-covalent in nature.

### 2.5. SUMO-Mediated Nuclear Localization of p38 Is Independent of p38 Phosphorylation

**Figure 7:**
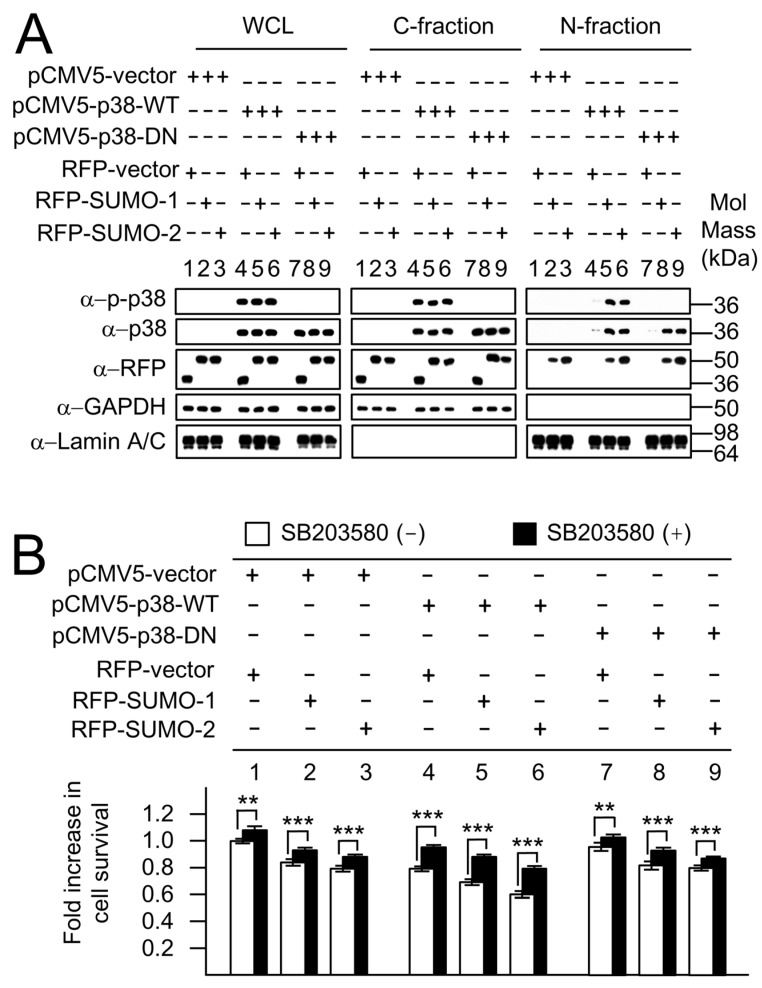
SUMOs mediate the nuclear transport of p-p38, p38-WT and p38-DN. AGS cells co-transfected with p38 proteins (wild type pCMV5-p38-WT and dominant negative pCMV5-p38-DN), and RFP-SUMOs were characterized for nuclear transfer of p38 and for SUMO-mediated p38 apoptotic pathway during *H. pylori* infection. (**A**) p38-WT and p38-DN were blotted with anti-p-p38 and anti p38 antibodies. Other antibodies used have been described in Figure 3. p38-DN was detected only using anti-p38 antibody, and not when anti-p-p38 antibody was used. p38 and p-p38 proteins were clearly observed in the nuclear fraction only when co-transfected with RFP-SUMO-1 and RFP-SUMO-2, and not distinctly when cotransfected with RFP alone, demonstrating that the nuclear localization is dependent on SUMOs. This effect was found to be independent of p38 phosphorylation as the nuclear localization of the phosphorylation sites mutant p38-DN was also dependent on the presence of SUMOs; and (**B**) p38 inhibitor (SB203580) was used in the MTT assay to show the effects of the increased levels of p38 and SUMOs in the p38 pathway. Compared with the basal state, p38-WT enhanced apoptosis (i.e., decreased cell survival), whereas p38-DN had no such function during *H. pylori* infection, highlighting the importance of p-p38, and consistent with p38-DN being a non-activatable (non-phosphorylatable) p38. The Western blots (**A**) were repeated three times and representative images are shown. The MTT assays (**B**) were repeated four times and the results shown are the mean ± standard deviation. Statistical analyses were performed using Student’s *t* test. *p* < 0.05 was considered significant; * *p* < 0.05; ** *p* < 0.01; *** *p* < 0.001.

The MAPK protein, p38, has previously been observed in both the cytoplasm and nucleus, but the transport mechanism into the nucleus was unclear. Previously, phosphorylation-dependent nuclear transfer of p38 has been suggested as the transport mechanism. However, our nuclear fractionation studies (Figure 3) showed that the nuclear localization of endogenous p38, similar to that of p-p38, is linked to the cellular levels of SUMOs. To examine the association between non-phosphorylated p38 and SUMOs, the pCMV5-p38-WT and pCMV5-p38-DN mammalian expression constructs were used. The roles of SUMOs in mediating nuclear location of p38-DN were further demonstrated using cellular fractionation assays (Figure 7A). The levels of p38-WT, p-p38-WT and p38-DN in C- and N-fractions were analyzed for RFP-vector, RFP-SUMO-1 and RFP-SUMO-2 co-expressing cells in response to *H. pylori* infection. RFP-vector (RFP) was used as a control for the RFP-fusion fragment without exogenous RFP-SUMO proteins. In contrast to the RFP/p38 co-expressing control cells (Figure 7A, lanes 4, 7), in RFP-SUMO-1/p38 and RFP-SUMO-2/p38 co-expressing cells, p-p38-WT (Figure 7A, lanes 5, 6) and p38-WT (Figure 7A, lanes 5, 6) as well as p38-DN (Figure 7A, lanes 8, 9) were all clearly observed in the N-fractions. These results are consistent with SUMO-mediated nuclear localization of p38 being independent of the p38 phosphorylation state.

A preliminary study on the apoptosis of AGS cells and GFP, wild type GFP-p38-WT and dominant negative GFP-p38-DN transfectants, incubated with or without *H. pylori* (300 MOI) for 12 h, stained with annexin-V were analyzed using flow cytometry as shown in Figure S4A. Results in Figure S4B showed that up-regulation of apoptosis was observed upon overexpression of p38-WT (group 3 vs. group 2) and in response to *Hp* infection (black bar vs. white bar). No upregulation of apoptosis was seen upon overexpression of p38-DN (group 2 vs. group 4).

The biological functions of phosphorylated p38-WT and non-phosphorylatable p38-DN were further analyzed using the MTT assay in p38/SUMO co-expressing cells during *H. pylori* infection (Figure 7B). In the absence of SB203580 p38 MAPK inhibitor (white bars), the results indicated that levels of cell survival in pCMV5-p38-DN transfectants (Figure 7B, groups 7–9) were similar to that of pCMV5-vector transfectants (Figure 7B, groups 1–3), whereas the levels of cell survival were decreased in pCMV5-p38-WT transfectants (Figure 7B, groups 4–6) when compared with pCMV5-vector control transfectants (Figure 7B, groups 1–3). Hence, the decrease in cell survival correlates with the presence of p-p38 in the nuclear fraction, particularly in the presence of SUMOs (groups 5 and 6) where high levels of nuclear p-p38 give rise to the lowest cell survival. In the case of the non-phosphorylatable p38 where there is no nuclear p-p38 (7A, N-fraction, Groups 7–9) the survival is not reduced (Figure 7B, bars 7–9) and is very similar to that for the pCMV-vector only control (Figure 7B; bars 1–3). These results demonstrate that the non-functional non-phosphorylated p38 (pCMV5-p38-DN), that cannot be phosphorylated in either the cytoplasm or in the nucleus, can be transferred by SUMOs but has no effect on cell survival; whereas the functional wild type pCMV5-p38-WT, that can be phosphorylated and can also be transferred by SUMOs is essential to exert an effect on viability. The results suggest that the nuclear transfer of p38 is SUMO-dependent, but independent of the phosphorylation state of p38, whereas p38-mediated apoptosis depends, as expected, upon nuclear p-p38. Cell survival was improved in all cases when cells were pretreated with SB203580 inhibitor (black bars) illustrating the importance of p38-mediated apoptosis. The decrease in cell-survival for the p38-WT cells was not completely reversed to the levels of the vector alone control, probably because of an incomplete block of the p38 pathway by the inhibitor. Similarly, the fact that the inhibitor exerts an effect on the cells lacking exogenous p-p38 (pCMV5 and pCMV5-p38-DN) suggests it is acting on endogenous p38 in these cases. Taken together, these results show that the effects of SUMOs on cell survival measured by the MTT assay reflect modulation of p38-dependent apoptosis through modulation of nuclear p-p38 levels.

Interestingly pull-down assays show that SUMO-2 binds better to p38 (phosphorylated or un-phosphorylated) than SUMO-1 does (Figure 4B and Figure 6A), although p38 and p-p38 are not covalently modified by SUMOs (Figure 6B). However, the direct interaction between SUMOs and p38 and p-p38 supports the hypothesis that the cytoplasmic SUMOs serve as carrier proteins for the nuclear transfer of p38 and p-p38 through a non-covalent interaction. The p38 and p-p38 found in the N-fraction of the control RFP/p38-WT co-expressing cells was minimal as a result of small amounts of endogenous SUMOs, in contrast to the larger levels of p38 and p-p38 found in the N-fraction of RFP-SUMOs/p38-WT co-expressing cells (Figure 7A). This highlights the importance of the expression levels of SUMOs for the nuclear relocation of p38 and p-p38 in response to *H. pylori* infection. The phosphorylation-dependent nuclear translocation of p38 has been suggested as a common phenomenon in response to various stresses. However, the results of our pull-down assays and nuclear fraction assays, suggest that the non-phosphorylatable p38-DN protein is associated with SUMOs and can be transferred to the nucleus by SUMOs (Figure 7). We now know that p38 and p-p38 are carried and transferred to the nucleus by SUMO-1 and SUMO-2 in the resting state (Figure 3). In contrast, in response to *H. pylori* infection it is the stress responsive SUMO-2 protein, with a stronger binding ability than SUMO-1 for p38 and p-p38, that serves as a major carrier to transfer p38 and p-p38 to the nucleus in response to *H. pylori* infection (Figure 3). This illustrates a new pathway for nuclear transfer of p38 through a non-covalent interaction with SUMOs that is independent of the p38 phosphorylation state. Moreover, in response to stress, not only p-p38 but also p38 are transferred to the nucleus by SUMOs, mostly by SUMO-2, in a SUMOs concentration-dependent manner.

Our pull-down assays and cellular fraction assays for SUMOs/p38 SIM mutants (Figure 4) showed that SIM3 (amino acids ^289^LVLD^292^) is the most important site for binding to SUMOs. To conclude, this study demonstrates non-covalent interactions between p38 and SUMOs, independent of the phosphorylation state of p38. A mechanism for p38 and p-p38 nuclear translocation by SUMO carrier proteins in response to *H. pylori* infection is proposed.

## 3. Discussion

As a highly conserved subfamily of MAPKs, p38 proteins are important mediators of cellular responses to a wide variety of environmental stresses, including bacterial lipopolysaccharide (LPS) [6], inflammation [1,2], ultraviolet radiation [7], and oxidative stresses [8]. In resting cells, p38 is distributed both in the cytosol and the nucleus, and cytosolic p38 proteins translocate into the nucleus in response to various stimuli in order to access their nuclear substrates. Thus, the intracellular redistribution of p38 is an important mechanism by which p38 fulfils its cellular functions. Although there are theories suggested for nuclear translocation upon activation for all MAPK family members, the mechanism of p38 nuclear translocation is still not known [5,6,7,8,9]. Since a typical NLS could not be identified in MAPKs, nor could direct interactions be detected between MAPKs and purified importins [9,32,33,34], it has been suggested that these proteins may undergo nuclear translocation either through interaction with other NLS-bearing proteins as carriers or, in the case of ERK, by a specific sequence that is designated as a *nuclear* translocation signal (*NTS*). However, no such sequence has been identified in p38 as yet [5] and the mechanisms of nuclear transport of p38 and p-p38 remain unclear. Our study suggests that SUMOs are important mediators of p38 nuclear import.

We found that *H. pylori* infection resulted in increased p38 activation and increased levels of SUMO-1 and SUMO-2 proteins, and that these increases were accompanied by p38-mediated cellular apoptosis. Manipulation of SUMO levels during *H. pylori* infection by either knockdown or overexpression, resulted in an increase or decrease in cell survival respectively. Hence, we proceeded to investigate whether a direct interaction between p38 and SUMOs mediates this SUMO-dependent p38 effect on viability following *H. pylori* infection.

Both the activation of p38 and nuclear localization of p38 and p-p38 were elevated significantly in RFP-SUMOs expressing cells, especially during *H. pylori* infection, further suggesting an association between SUMOs and p38. Furthermore, the results of the pull-down assays clearly demonstrated interactions between SUMOs and p38. In the absence of *H. pylori* infection both SUMO-1 and SUMO-2 contributed to the p38 nuclear localization, whereas in the presence of *H. pylori* only SUMO-2 was found to play this role. SUMOylation assays demonstrated that p38 is not covalently modifiable by SUMOs so we concluded that SUMOs mediate nuclear import by binding to p38/p-p38 through non-covalent interactions. The nature of this interaction was clarified by investigation of p38 SIM mutants, and results from pull-down and cellular fractionation assays showed that the SIM3 sequence (amino acids ^289^LVLD^292^) is the key mediator of the non-covalent interaction with SUMOs. Other studies have recently highlighted the biological importance of non-covalent SUMO-SIM interactions [35,36] e.g., a C-terminal SIM in the serine/threonine kinase HIPK2 is essential for its localization to nuclear speckles and its uptake in PML-NBs, while a SIM in the anti-recombinogenic helicase Srs2 is essential for its interaction with SUMOylated PCNA (a DNA polymerase δ processivity factor essential for homologous recombination).

The SUMO-mediated nuclear transport of p38 occurred equally well for wild type or for an inactive dominant negative form of p38 that could not be phosphorylated. We conclude that SUMO-2 is an important mediator of p38 nuclear transport in response to *H. pylori* infection via a non-covalent mechanism of binding that is independent of the p38 phosphorylation state, and that the SUMO-2-p38 interaction plays a key role in cell survival following *H. pylori* infection. It will be interesting to discover whether SUMO plays a similar role in the cellular response to other stressors.

## 4. Materials and Methods

### 4.1. Cell Culture and H. pylori Infection

Gastric epithelial cell lines AGS (BCRC) were grown in RPMI-1640 and purchased from Thermo Fisher Scientific (Waltham, MA, USA) The *H. pylori* strain ATCC 43504 (*CagA*^+^, *VacA*^+^) was maintained on CDC ANA blood agar (BD Biosciences, Franklin Lakes, NJ, USA) and resuspended in phosphate-buffered saline (PBS, Life Technologies, Carlsbad, CA, USA) [37]. *H. pylori* were added to cells at a ratio of 300:1 bacteria to cells [18].

### 4.2. p38 SUMO-Interacting Motif (SIM) Prediction and Production of Full Length p38-SIM Mutant Constructs

We divided p38α (MAPK14) into fragment-1 (1–110 amino acids), fragment-2 (111–180 amino acids) and fragment-3 (181–360 amino acids) (Figure 4). The pull-down assay showed that these fragments all interacted with SUMOs [31]. The highest probability putative SIMs predicted within these fragments by JASSA (http://www.jassa.fr/) and GPS-SBM 1.0 (http://sbm.biocuckoo.org/) are ^83^VIGL^86^ (named p38-SIM1) in fragment-1, ^164^LKIL^167^ (p38-SIM2) in fragment-2 and ^289^LVLD^292^ (p38-SIM3) in fragment-3. Predicted SIMs from three SUMO-p38 interacting fragments were individually mutated in the context of full-length p38 to give three full length p38-SIM mutants (p38-SIM1*, p38-SIM2* and p38-SIM3*) with two amino acid residues mutated in each. Polymerase chain reaction (PCR) fragments of p38-SIM1*, p38-SIM2* and p38-SIM3* coding sequences were then created through overlap extension PCR, using pCMV5-p38-WT as a template, and cloned in pGEX-KG and pCMV5.

### 4.3. Plasmids

The p38α coding sequence was assembled by ligation of PCR fragments in the mammalian expression vector pCMV5 to make the wild type p38 construct (pCMV5-p38-WT). The dominant negative p38 construct (pCMV5-p38-DN) was created through site-direct mutagenesis using the QuikChange mutagenesis kit (Agilent) with pCMV5-p38-WT as a template. The PCR fragments of pCMV5-p38-WT and pCMV5-p38-DN coding sequences were ligated to the *Escherichia coli* (*E. coli*) expression vector pGEX-KG to create glutathione-*S*-transferase-tagged GST-p38-WT and GST-p38-DN. The construction of pDsRed1-C1 (RFP, Clontech, Mountain View, CA, USA) fused plasmids for active SUMOs (RFP-SUMO-1 and RFP-SUMO-2) and GST-Daxx4 (a GST fusion with the 607–740 aa tail fragment of Daxx) was previously described [18]. All plasmid sequences were checked by sequencing analysis.

### 4.4. Reverse Transcription Polymerase Chain Reaction (RT-PCR)

For cDNA synthesis 1 μg of each RNA sample was reverse-transcribed. PCR products in agarose (Amresco, Solon, OH, USA) gels, visualized by ethidium bromide (EtBr, Sigma-Aldrich, St. Louis, MO, USA) staining were digitally photographed for intensity analysis.

### 4.5. Transfections

AGS cells were transfected using Lipofectamine 2000 (Life Technologies). Briefly, 4 μL lipofectamine 2000 was mixed with 1.6 μg of DNA in OPTI-MEM medium (Life Technologies) and added to approximately 1 × 10^6^ cells. After 2 h, the medium was removed and cells were cultured in fresh medium supplemented with 10% FBS. The transfectants were analyzed by Western blotting and MTT assay.

### 4.6. Western Blots

AGS cell whole cell lysates were made as follows: AGS cells were harvested and resuspended in lysis buffer (1% Triton X-100 (Acros), 50 mM Tris (pH7.9) (Merck Millipore, Burlington, MA, USA), 5 mM EDTA (Sigma-Aldrich), 50 mM NaF (Fluka), 0.1 mM Na_3_VO_4_ (Sigma-Aldrich), 50 mM *N*-ethylmaleimide (NEM, Sigma-Aldrich) and 1% protease inhibitor cocktail (Sigma-Aldrich)) on ice for 10 min. Western blotting was conducted as described previously [18].

### 4.7. Antibodies

Antibodies used in this study included rabbit anti-phospho-p38 (Thr180/Thr182, #9211, Cell Signaling Technology, Danver, MA, USA), rabbit anti-p38 (#9212, Cell signaling), mouse anti-GAPDH (MAB374) (Merck Millipore, Burlington, MA, USA), mouse anti-GMP1(SUMO-1) (18-2306, Life Technologies), rabbit anti-SUMO-2/3 (BML-PW9465, Enzo Life Sciences, Farmingdale, NY, USA), rabbit anti-lamin A/C (#2032, Cell Signaling), rabbit anti-RFP (632496, Clontech, Mountain View, CA, USA), rabbit anti-GST (71-7500, Life Technologies), rabbit anti-His (SC-803, Santa Cruz Biotechnology, Dallas, TX, USA), horseradish peroxidase (HRP)-conjugated goat anti-mouse IgG (G21040, Life Technologies) and HRP-conjugated goat anti-rabbit IgG (G21234, Life Technologies). The rabbit anti-phospho-p38 and the rabbit anti-p38 used in this study can both react with the various isoforms of p38 (p38α, p38β, p38γ, and p38δ). However, the main isoform expressed in AGS cells is p38α, so this is referred to simply as p38 throughout the paper.

### 4.8. MTT Assay

The colorimetric MTT assay was used to assess cell viability as follows. AGS cells (2 × 10^5^ cells) were transfected with expression vectors for 24 h, then treated with SB203580 MAPK inhibitor (10 μM, Merck-Millipore, Burlington, MA, USA) for 1h prior to *H. pylori* infection for 12 h. 3-(4,5-Dimethylthiazol-2-yl)-2,5-diphenyltetrazolium bromide (MTT) (12 mM, Amresco, Solon, OH, USA) was added and incubated in the dark at 37 °C for 4 h. DMSO was added to dissolve the formazan crystals and read by spectrophotometer (BioTek, Winooski, VT, USA). The results were calculated as percentages of the control (vehicle).

### 4.9. In Vitro p38 Phosphorylation

It is interesting to note that the ATP regeneration system in in vitro SUMOylation reactions could autophosphorylate GST-p38-WT (Figure 6B, group 1), as revealed by blotting with anti-p-p38 antibodies; however, autophosphoryation did not occur at the dominant negative phosphorylation site mutant GST-p38-DN (Figure 6B, group 2). Therefore, this system was used to produce phosphorylated GST-p38-WT (GST-p-p38-WT) to enable us to examine the binding affinity between SUMOs and p-p38 in the pull-down assays.

Reaction mixtures containing an ATP regeneration system [38] and GST-p38-WT proteins, were incubated for 3 h at 37 °C prior to in vitro pull-down assay.

### 4.10. In Vitro Pull-Down Assay

The in vitro pull-down assay was used to determine whether there was a physical interaction between 6-His tagged SUMOs and GST tagged p38 wild type or mutant proteins. It was performed by using 6× His-SUMOs (1–5 μg) immobilized onto Ni-NTA Magnetic Agarose Beads (Qiagen, Hilden, Germany) along with GST fusion proteins (1 μg) in 500 μL of binding buffer [39] for 2 h at room temperature. The beads were washed with wash buffer five times prior to elution of the bound proteins with SDS sample buffer and analysis by Western blotting.

### 4.11. In Vitro SUMOylation Assay

Recombinant GST and His fusion proteins were produced in *E. coli* strain BL21 and purified by standard methods. Each reaction sample contained an ATP regeneration system [38], GST-SAE1/2, GST-Ubc9, His-SUMO-1 or His-SUMO-2, and substrates of GST, GST-p38-WT, GST-p38-DN or GST-Daxx4. GST-Daxx was used as a positive control as it has previously been observed that GST-Daxx was strongly modified by SUMO-1 and weakly modified by SUMO-2. The reactions were incubated at 37 °C for 3 h, then stopped by adding 1/5 vol. 6× SDS sample buffer, boiled and separated by SDS-PAGE. The SUMOylation products were analyzed by Western blotting.

### 4.12. Nuclear and Cytosolic Isolation

AGS cells were harvested and resuspended in cytosolic lysis buffer (0.1% NP40 (Amresco), 10 mM Tris (pH7.9), 10 mM MgCl_2_ (Sigma-Aldrich), 15 mM NaCl, 50 mM NEM, and 1% protease inhibitor cocktail) on ice for 10 min. After brief centrifugation, the supernatant was saved as the cytosolic fraction, and the nuclear pellet was lysed in SDS sample buffer. The cytosolic and nuclear fractions were analyzed by Western blotting.

### 4.13. Statistical Analyses

Differences between groups were evaluated by an independent Student’s *t*-test using SPSS version 20.0 (SPSS Inc., Chicago, IL, USA). *p* < 0.05 was considered significant

## Supplementary Materials

Supplementary materials can be found at http://www.mdpi.com/1422-0067/19/9/2482/s1.

## Abbreviations

*H. pylori*	*Helicobacter pylori*
MAPK	Mitogen-activated protein kinase
MOI	Multiplicity of infection
SIM	SUMO interacting motif
SUMO-1	Small ubiquitin-like modifier-1
SUMOs	Small ubiquitin-like modifier-1 or 2
