# Supplementary material for: SUMOs Mediate the Nuclear Transfer of p38 and p-p38 during Helicobacter Pylori Infection
Source: Int J Mol Sci. 2018 Aug 22;19(9):2482. doi: 10.3390/ijms19092482 (PMC6163533; doi:10.3390/ijms19092482)
Supplement: Supplementary file 1 [file ijms-19-02482-s001.zip › ijms-333188-supplementary.docx]

Supplementary Materials

SUMOs Mediate the Nuclear Transfer of p38 and
p-p38 During *Helicobacter Pylori* Infection

**
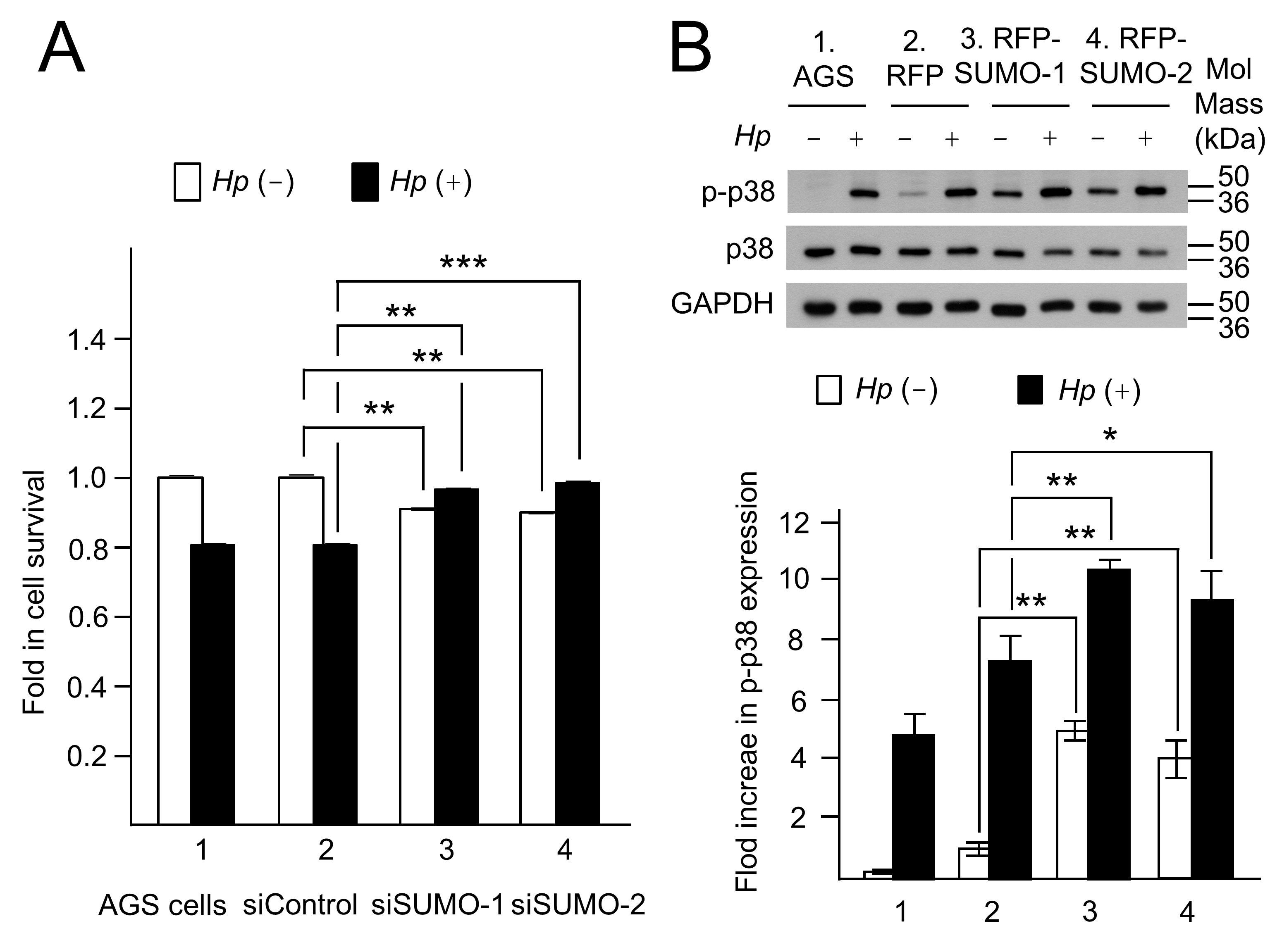
**

**Figure S1.** The effects of *Hp* infection on cell survival and on the levels of p-p38 expression. (**A**) *H. pylori* induced apoptosis is associated with SUMOs. The levels of cell survival for various transfectants (siControl, siSUMO-1 and siSUMO-2) without *H. pylori* infection (white bars) or with *H. pylori* infection (black bars) were examined in AGS cells using MTT assays. In the absence of *H. pylori* infection a reduction in cell survival was observed in siSUMO-1 and siSUMO-2 transfectants compared with siControl (white bars in groups 3 and 4 compared with that in group 2). However, *H. pylori* infection induced a reduction in cell survival in untransfected and siControl transfected AGS cells (white bars compared with black bars in groups 1 and 2). The knock down of SUMO-1 (group 3 siSUMO-1) and SUMO-2 (group 4 siSUMO-2) up-regulated cell survival in *H. pylori* infected cells compared with siControl transfectants (black bars in groups 3 and 4 compared with black bar in group 2). These results show that endogenous SUMOs mediate the *H. pylori* induced apoptosis. The MTT assays were repeated four times and the results shown are the mean +/− standard deviation. Statistical analyses were performed using Student’s *t* test. *p* < 0.05 was considered significant; * = *p* < 0.05; ** = *p* < 0.01; *** = *p* < 0.001; * = *p* < 0.05; ** = *p* < 0.01; *** = *p* < 0.001. (**B**) Overexpression of SUMOs enhance endogenous p38 phosphorylation. RFP-SUMO-1 and RFP-SUMO-2 transfectants were incubated for 12 h before being infected with *H. pylori* (300 MOI, 45 min) and the levels of phosphorylated p38 were analyzed by Western blot (top panel). Quantification of the bands in the Western blot (B; lower panel) showed that *H. pylori* infection resulted in increased p-p38 compared with uninfected cells. Significant increases in phosphorylated p38 (p-p38) were observed both in RFP-SUMO-1 and RFP-SUMO-2 overexpression cells (groups 3 and 4 respectively) versus RFP (group 2) with or without *H. pylori* infection. The *p* values obtained for the data in Supplementary Figure 1A,B are summarized in Supplementary Table 1. The Western blots were repeated three times and representative images are shown (top panel of (**B**)).

**
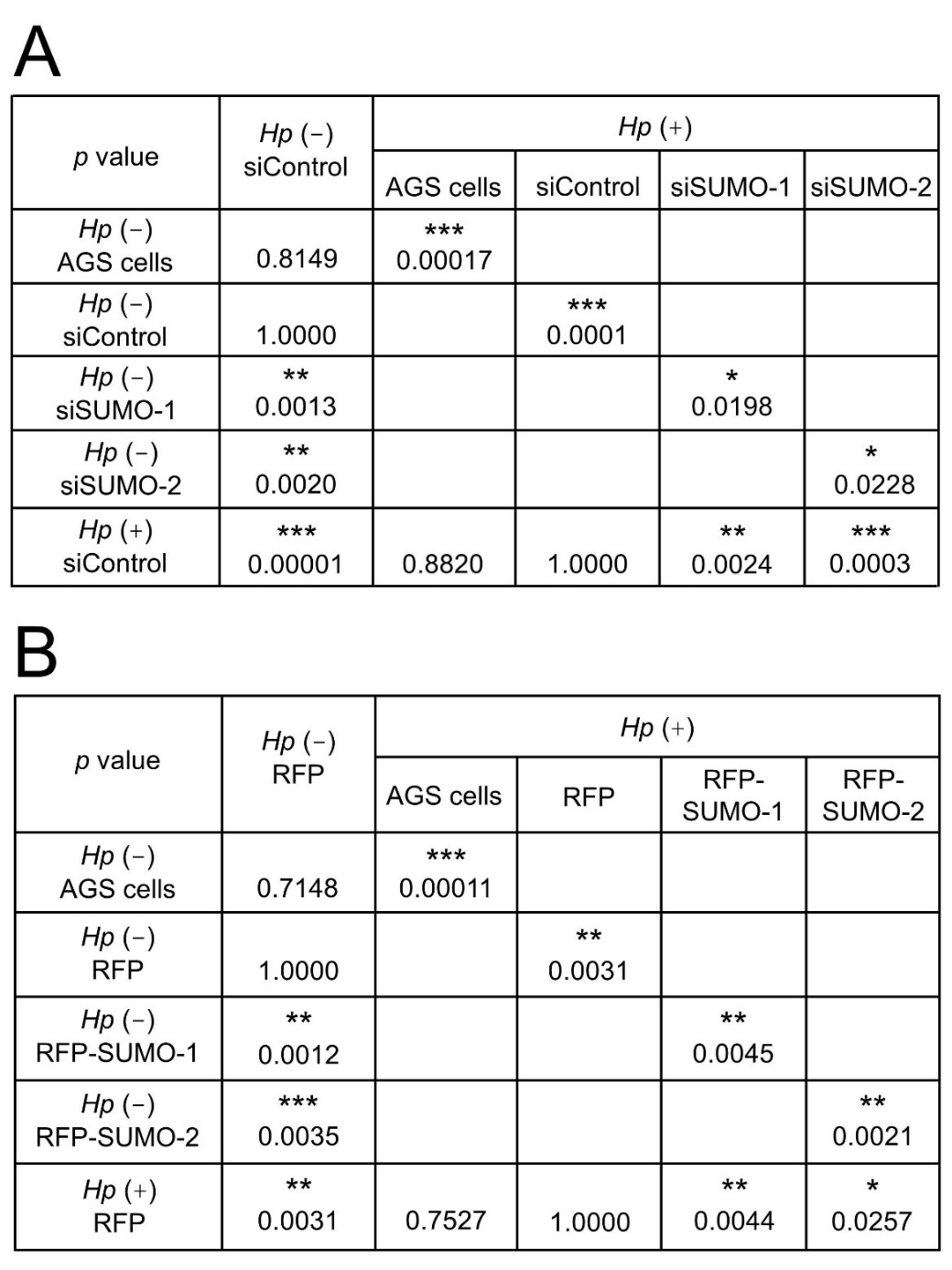
**.

**Table S1.** Summary of the p values for statistical analyses for comparison of the data from Supplementary Figure 1 with (+) and without (−) *H. pylori* infection (**A**) *p* values from the MTT assays (Supplementary Figure 1A) used to assess the effects on cell survival of knock down of SUMOs using siSUMO-1 and si-SUMO-2; and (**B**) *p* values from quantification of Western blots (Supplementary Figure 1B) used to assess the changes in phosphorylated p38 resulting from overexpression of SUMOs, using RFP-SUMO-1 and RFP-SUMO-2. Statistical analyses were performed using Student’s *t* test. *p* < 0.05 was considered significant; * = *p* < 0.05; ** = *p* < 0.01; *** = *p* < 0.001.


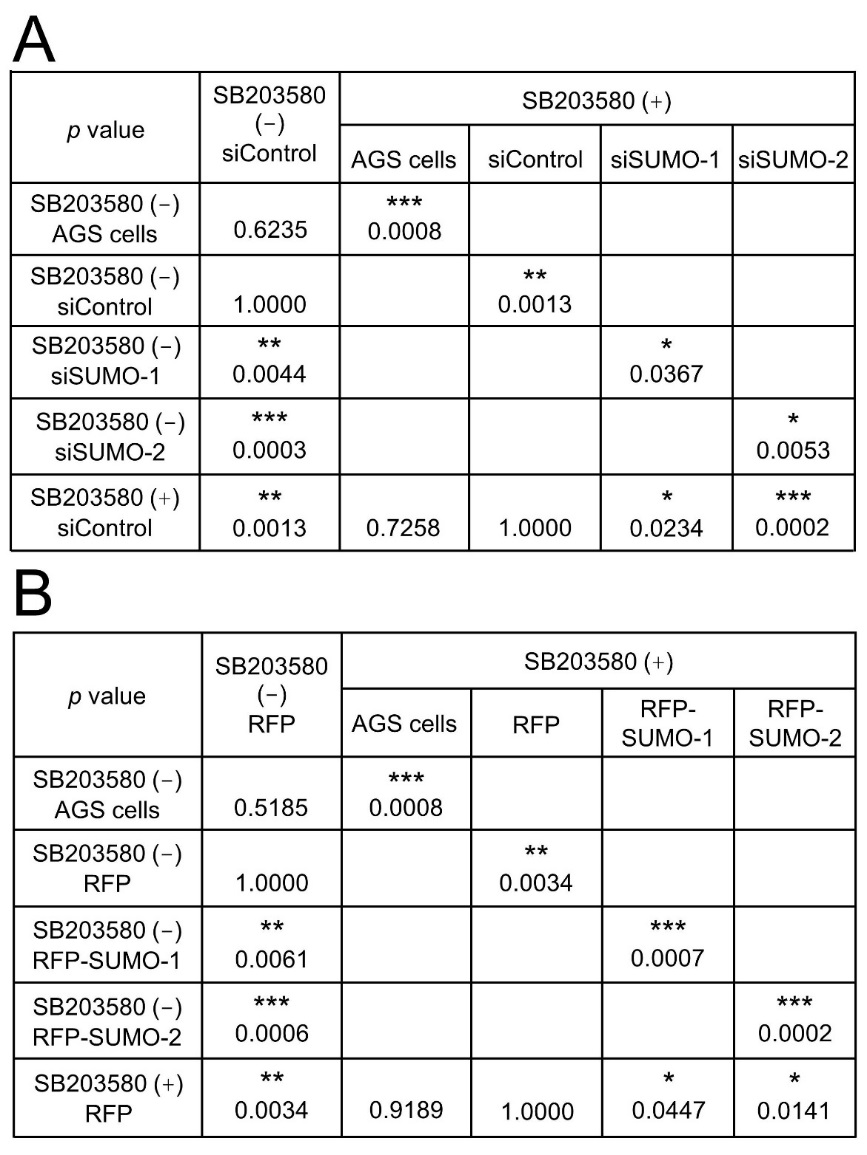


**Table S2.** Summary of the p values for statistical analyses for comparison of the data with and without the p38 MAPK inhibitor SB203580 from Figure 2. The data are shown for SB203580 (+) vs. SB203580 (−) conditions for (**A**) experiments to knock down SUMOs using siSUMO-1 and si-SUMO-2 and (**B**) experiments where SUMOs were overexpressed, using RFP-SUMO-1 and RFP-SUMO-2, as shown in the lower panels of Figure 2A,B. Statistical analyses were performed using Student’s *t* test. These results show that the increase in cell survival in the presence of the inhibitor compared with its absence is significant for all pairwise comparisons. *p* < 0.05 was considered significant; * = *p* < 0.05; ** = *p* < 0.01; *** = *p* < 0.001.


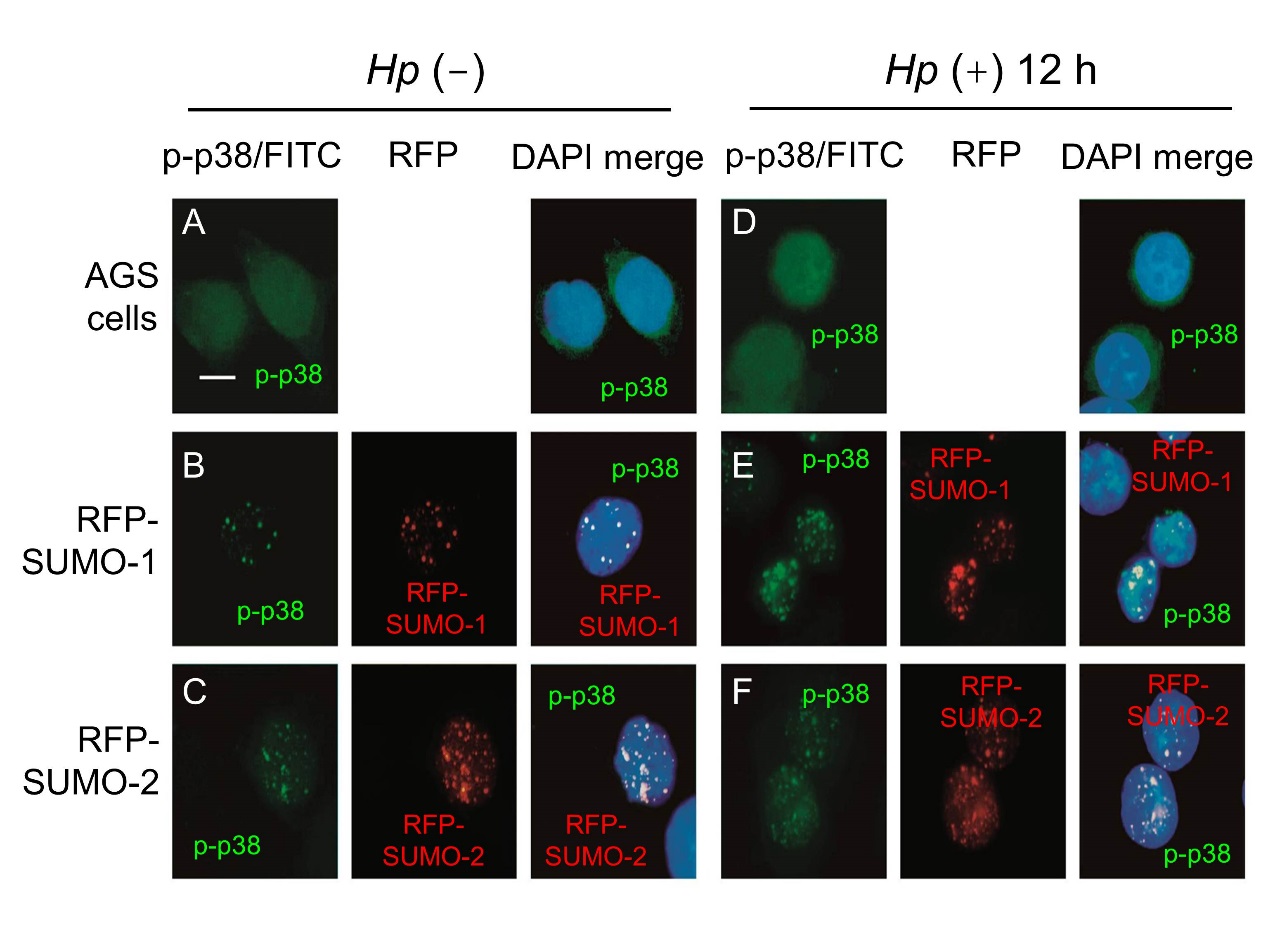


**Figure S2.** Endogenous p-p38 co-localized with RFP-SUMOs in the nucleus and its levels were increased during *Hp* infection. The cellular localization of phosphorylated p38 was analyzed using p-p38/FITC antibodies in AGS cells and RFP-SUMO-1 and RFP-SUMO-2 transfected cells that were incubated for 12 h before being infected with *H. pylori* (300 MOI, 12 h). In untransfected AGS cells dispersed endogenous nuclear p-p38 was observed in both non-infected cells (Panel A) and *Hp* infected cells (Panel D). Comparison of panel A and D showed that nuclear p-p38 was higher in the *Hp* infected cells. In RFP-SUMOs overexpressing cells p-p38 co-localized with RFP-SUMOs in nuclear dots both in non-*Hp* infected cells (group B and group C) and in infected cells (group E and group F). p-p38 was increased during *Hp* infection (group E vs. group B, and group F vs. group C). All experiments were repeated three times and representative images are shown.

**
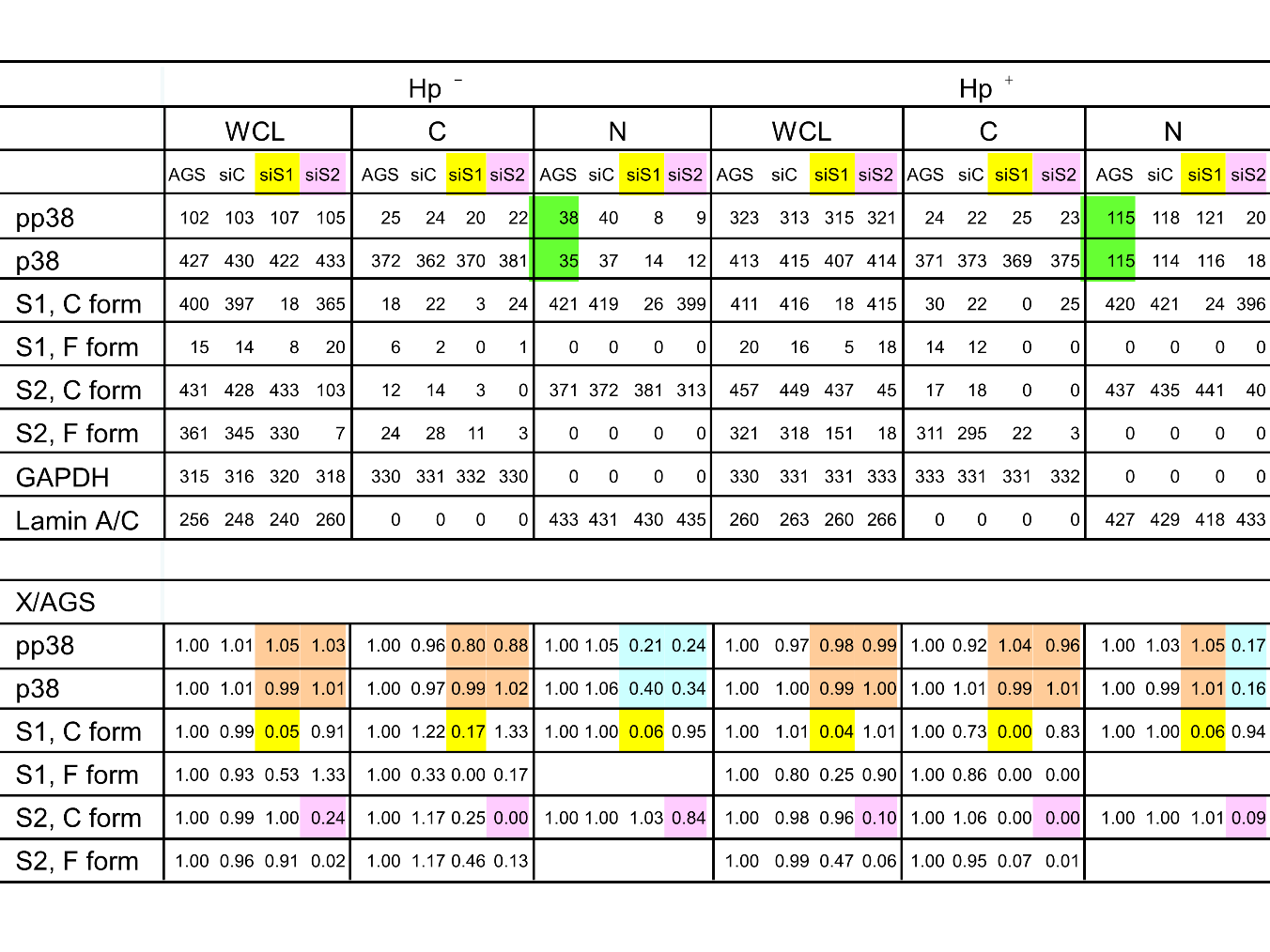
**

**Table S3.** Quantification of the Western blot data shown in Figure 3A. The upper panel shows the values for the quantification of the bands for *Hp*^−^ and *Hp*^+^ conditions. The values highlighted in green show the increase in nuclear p38 and p-p38 for *Hp*^+^ compared with *Hp*^−^ conditions. The lower panel shows the values after normalization of the results to those for the untransfected AGS cells in each fraction. These values show the down-regulation of the SUMO-1 and SUMO-2 after transfection of siSUMO-1 and siSUMO-2 (yellow and pink blocks), and the effect on p38 levels (brown and blue blocks). The brown blocks highlight conditions with little or no effect on p38 while blue blocks show conditions giving major reductions in p38 or p-p38 levels. In the absence of *H. pylori* infection p38 and p-p38 were decreased in both siSUMO-1 and siSUMO-2 transfectants. However, during *H. pylori* infection the nuclear levels of p38 and p-p38 only decreased in siSUMO-2 transfectants.


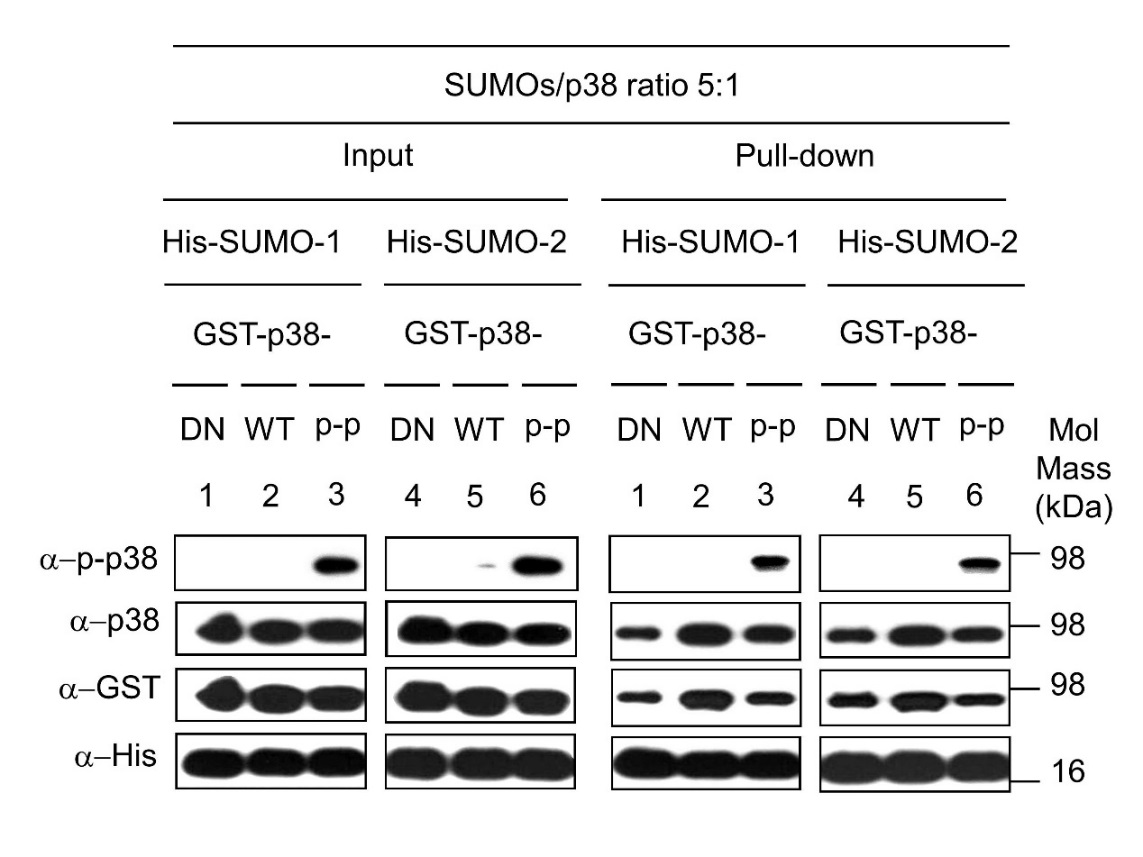


**Figure S3.** Both SUMO-1 and SUMO-2 bind to p38-DN, p38-WT and p-p38 at a high SUMO to p38 ratio. The pull down assay conditions were the same as for Figure 6 except a SUMO to p38 ratio of 5:1 (rather than 1:1) was used. The blots for input proteins and the pull down proteins were probed with antibodies as indicated. These results show that at the higher SUMO to p38 ratios SUMO-1 can pull-down GST-p38-DN, GST-p38-WT and GST-p-p38 in a similar manner to SUMO-2. This is in contrast with the results at the lower SUMO to p38 ratio (1:1 in Figure 6) where only SUMO-2 gave efficient pulldowns. All experiments were repeated three times and representative images are shown.


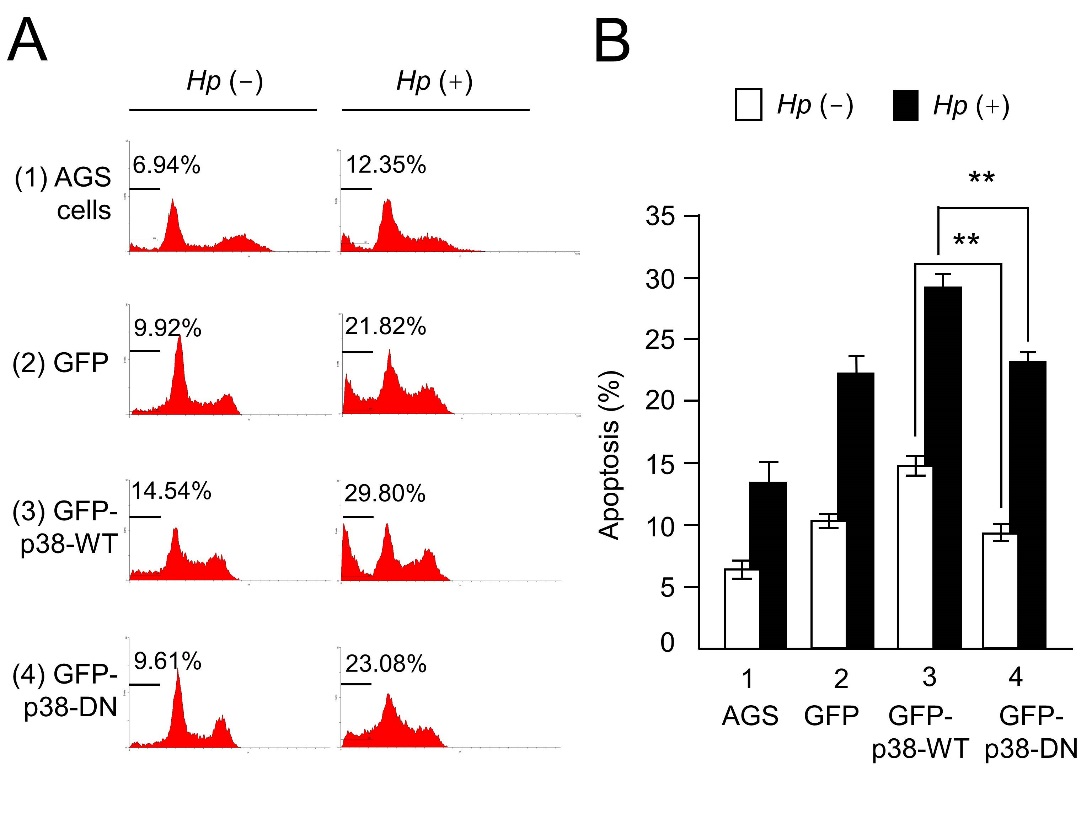


**Figure S4.** Up-regulation of apoptosis in response to *Hp* infection. AGS cells and GFP, wild type GFP-p38-WT and dominant negative GFP-p38-DN transfectants were incubated with or without *H. pylori* (300 MOI) 12 h. (**A**) The apoptotic cells stained with annexin-V were analyzed using flow cytometry in three separate experiments and representative traces from one such experiment are shown. (**B**) The percentages of apoptosis increased in *H. pylori* infected cells (black bars) in comparison to the non-infected cells (white bars). Results shown are the mean +/− standard deviation for three experiments. The levels of apoptosis in the wild type GFP-p38-WT overexpression cells (group 3) were significantly increased in comparison to the dominant negative GFP-p38-DN overexpression cells (group 4) in both *H. pylori* infected and non-infected cells. There was no increase in apoptosis for GFP-p38-DN transfectants compared with the GFP control transfectants.
